# Primary ciliary dyskinesia diagnosis and management and its implications in America: a mini review

**DOI:** 10.3389/fped.2023.1091173

**Published:** 2023-09-08

**Authors:** M. Castillo, E. Freire, V. I. Romero

**Affiliations:** School of Medicine, Universidad San Francisco de Quito, Quito, Ecuador

**Keywords:** kartagener syndrome, Latin America, ciliopathies, cilia, Ecuador, PICADAR

## Abstract

**Introduction:**

Primary ciliary dyskinesia (PCD) is a rare genetic disorder that can result in significant morbidity and mortality if left untreated. Clinical manifestations of PCD include recurrent respiratory infections, laterality defects, and infertility, all of which arise from impaired or absent ciliary motility. Diagnostic approaches for PCD may include high-speed video microscopy, measurement of nasal nitric oxide levels, and genetic testing; however, no single definitive diagnostic test exists. The present study aims to highlight the lack of standardized diagnostic and treatment guidelines for PCD in Latin America (Central and South America, and the Caribbean). To this effect, we compared North American and European recommendations for the diagnosis and management of PCD and found that certain diagnostic tools and treatment options mentioned in these guidelines are not readily accessible in many Latin American countries.

**Methods & Results:**

This review gathers disease information in North America, Europe, and Latin America organizing guideline results into tables for clarity and potential interventions. Management information for Latin America is inferred from case reports, as most findings are from North American recommendations and studies on PubMed, Google Scholar, and Scopus. Treatment and management information is based on North American and European standards.

Among 5,774 publications reviewed, only 15 articles met the inclusion criteria (focused on PCD management, peer-reviewed, and located in America). No clinical guideline for PCD in Latin America was found, but recommendations on respiratory management from Colombia and Chile were discovered. The lack of guidelines in Latin America may originate from limited resources and research on the disease in those countries.

**Discussion:**

PCD lacks documentation, research, and recommendations regarding its prevalence in Latin America, likely due to unfavorable economic conditions. This disadvantage results in limited access to diagnostic tests available in North America and Europe. The PICADAR score, discussed in this review, can be used in low-income nations as a screening tool for the disorder.

## Introduction

### Definition and epidemiology

Primary ciliary dyskinesia (PCD) is a rare inherited disease, mostly autosomal recessive or, scarcely, X-linked. It is characterized by immotile, absent, or dysmotile cilia (1, 2).

PCD is classified as a rare condition, with an estimated incidence ranging from one in 10,000 to 40,000 individuals (3). However, this estimate is likely an underestimation due to frequent misdiagnosis, limited access to diagnostic facilities, and the tendency to mistake its symptoms for those of other conditions (4). The prevalence of this illness is uncertain as most of the available data is limited to North America, with a lack of epidemiological studies in Latin America, as well as insufficient medical recommendations and resources for diagnosis and therapy (1).

According to a European survey, PCD is typically diagnosed around the age of 5.3 years, and there is a significant correlation between delayed diagnoses and overall healthcare expenditures by the government (5).

The incidence of PCD is often underestimated due to a lack of awareness and diagnostic facilities, particularly in developing countries. It is crucial to consider that siblings of PCD patients can be carriers; thus, screening in family members should be performed (6, 7). Additionally, it is worth noting that 80% of neonates with PCD exhibit respiratory distress within the first 12 h of life (2).

### Pathophysiology and clinical manifestations

PCD is an inherited disorder characterized by the impaired motility of cilia, which plays a crucial role in the optimal functioning of the respiratory pathways and reproductive system. Motile cilia are specialized organelles located on the apical epithelial surface of the upper and lower airways. These cilia have a unique anatomical and functional structure, with dynein arms extending from the outer doublet A microtubules and attaching to the adjacent B microtubule. Additionally, they possess a central microtubular pair, resulting in the characteristic “9 + 2” arrangement observed in transmission electron micrographs (6).

The assembly and function of these motile cilia are meticulously regulated by numerous genes involved in the synthesis of microtubule-associated proteins. Genetic mutations affecting these genes can lead to impairment of mucociliary clearance throughout the respiratory tract (7–9). Consequently, this ciliary dysfunction manifests as a wide range of clinical presentations in children affected by PCD, typically including neonatal respiratory distress, chronic sinusitis and otitis media, hearing impairment, persistent coughing, recurrent respiratory tract infections.

Spermatozoa flagella has a central structure called the axoneme which is an arrangement of nine doublet microtubules (DM) and a central pair thus a 9 + 2 structure, outside of the DM there also are dynein as in motile cilia of the respiratory tract. Mutations in these dynein lead in some cases to infertility (6, 7, 10).

### Diagnosis

PCD exhibits clinical characteristics that can overlap with other conditions, such as primary immunodeficiencies, cystic fibrosis, asthma, and respiratory tract infections (8). Therefore, it is crucial to understand the clinical features of PCD and the available diagnostic tools.

Currently, there is no specific gold standard test for PCD diagnosis. Accurate diagnosis requires a combination of multiple investigations, including gene testing panels which analyze an approximate of 40–47 genes known for PCD (8, 9, 11).

European and North American guidelines recommend the use of high-speed video microscopy (HSVM) and nasal nitric oxide testing (8, 9). Furthermore, ALI (Air-Liquid Interface) culture can be used as a diagnostic aid in which nasal brush and bronchial epithelium biopsies are taken and show reduced ciliary beat frequency which facilitates diagnosis or exclusion of PCD (12).

Transmission electron microscopy (TEM) is another diagnostic tool that aids in identifying ciliary ultrastructural defects. Biopsies taken from nasal or bronchial mucosa for both TEM and HSVM can reveal cilia immobility, partial loss, or absence of the central pair of the cilia structure (8, 9).

In developing countries, the PICADAR score, which is based on clinical characteristics, serves as an alternative method to identify patients who require further testing and have a higher probability of PCD diagnosis. The presence of a wet cough is necessary for applying the PICADAR score system, which has a sensitivity of 90% and specificity of 75% (6, 13). A full score of 14 points corresponds to a 99.80% probability of having PCD, a score of ≥10 indicates a 92.6% probability, and a score of ≥5 indicates an 11.10% probability (13).

### Treatment and management

There is currently no established gold standard treatment for PCD, and therapies often draw from those used for cystic fibrosis and non-CF bronchiectasis. The current treatment approach includes physiotherapy, antibiotic administration, and the avoidance of risky behaviors or exposures (2, 14).

Respiratory tract infections in PCD are commonly caused by pathogens such as Pseudomonas aeruginosa, Streptococcus pneumoniae, Staphylococcus aureus, and Haemophilus influenzae (7, 9). Non-tuberculous mycobacteria (NTM) is found in 9%–18% of patients. Macrolides are recommended as antibiotic therapy in the absence of NTM. Low doses of azithromycin may help reduce exacerbations, although more research is needed specifically for PCD. The use of inhaled gentamicin is a topic of debate but has demonstrated efficacy in bronchiectasis (see Supplementary Table S1) (2, 8).

Manifestations of PCD include sinus infections, which can be treated with nasal steroids, nasal lavage, antibiotics, and, in some cases, surgery for polyp removal. Nebulized 7% hypertonic saline has been shown to improve lung function (2, 5). Otitis media with otorrhea may require the insertion of tympanostomy tubes. Lung transplant is an option for end-stage PCD, while functional endoscopic sinus surgery (FESS) is considered in cases of severe chronic sinusitis (15, 16).

Prevention plays a crucial role and involves regular vaccinations (such as influenza and pneumococcal vaccines), follow-up appointments, and lung function monitoring every 6–12 months. Promoting physical activity can help improve respiratory muscle strength and lung health in patients with obstructive pulmonary disease (8, 9).

## Methods & results

A search was conducted in June and July 2022 using various databases including PubMed, SCIELO, SCOPUS, and Google Scholar. Access to the paid databases was supported by the Universidad San Francisco de Quito digital library. The search algorithm utilized terms related to the Americas, Carribean, Latin America, Primary Ciliary Dyskinesia, PCD, Kartagener, and associated terms in both English and Spanish. The focus was on papers published after 2015 to capture recent recommendations, and exclusion criteria were applied to limit the number of publications.

**Figure 1:**
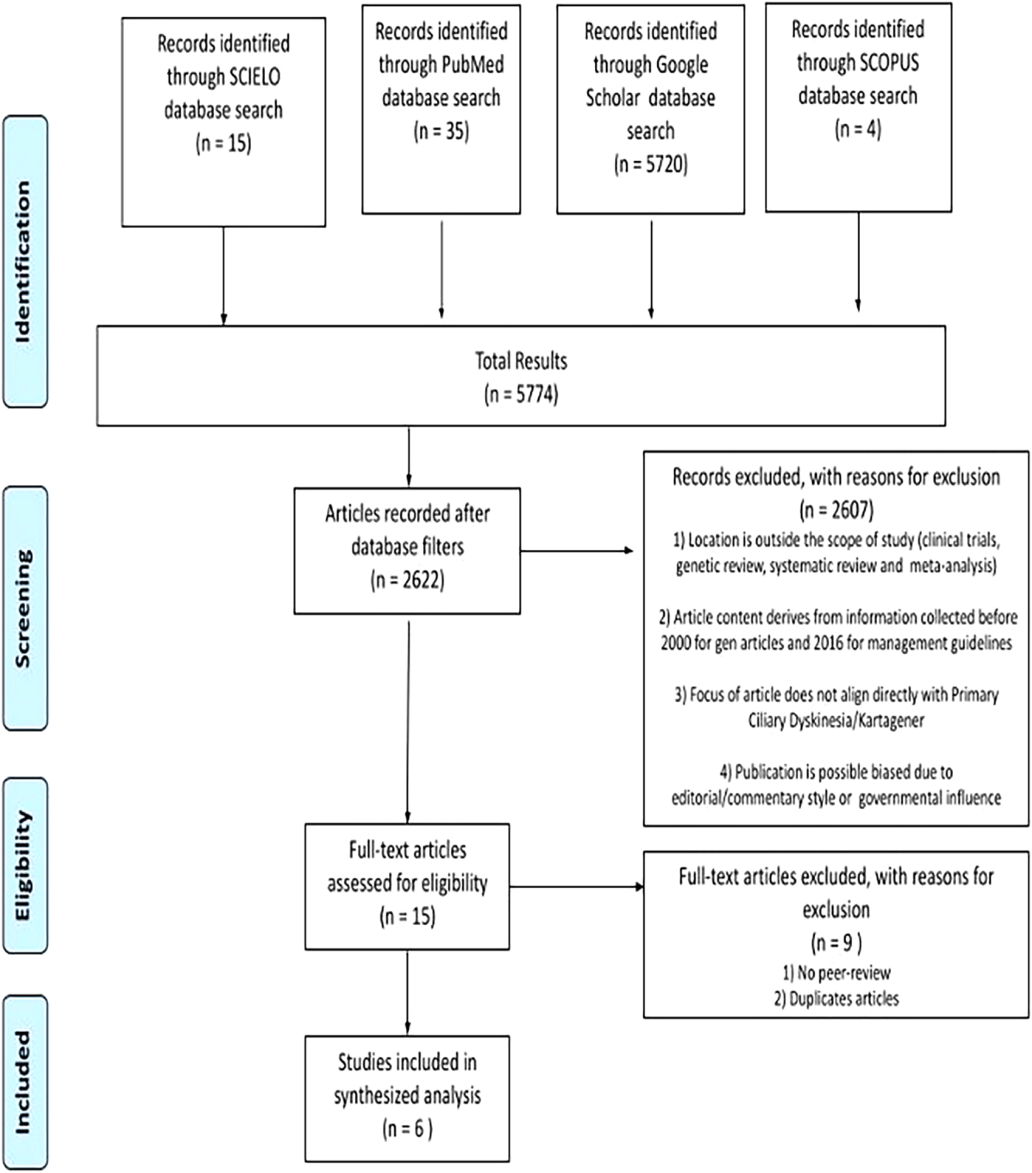
PRISMA flow diagram for search to investigate PCD.

The requirements for inclusion in the study were relevance to PCD management, location in the Americas, exclusion of publications from UR Entities or the government, and peer-reviewed status. All papers identified in the databases underwent a thorough title and abstract review based on the inclusion and exclusion criteria. Relevant information such as title, abstract, authors, and screening results were recorded, along with the article URLs. Only peer-reviewed journal articles were retained after the initial screening process. The data obtained from the selected articles was used to construct the Prisma diagram (Figure 1) to illustrate the flow of the study selection process.

Initially, 5,774 publications were identified using the algorithm. After applying the location and year filters, 2,622 articles remained. These papers underwent detailed examination and assessment based on the specified criteria. Ultimately, only fifteen articles met the prerequisites and inclusion criteria. Following the final screening, six papers were retained, considering peer review and the removal of duplicate articles. The information from all these articles is displayed in Table 1.

**Table 1:** Articles that met all the criteria in the research with a summary of the data about management in PCD.

Title	Year of publication	Author	Location	Therapy conclusions	
Diagnosis, monitoring, and treatment of primary ciliary dyskinesia: PCD foundation consensus recommendations based on state-of-the-art review (19)	2016	Adam J Shapiro, Stephanie D Davis, Deepika Polineni, et. all	Canada/North America	-Management should involve a multidisciplinary team. In routine therapies, it is highly recommended airway clearance through daily physiotherapy. -Hyperosmolar agents have not been studied and unclear long-term benefits have been shown, mannitol has no studies. -At least 2−3 weeks of broad-spectrum oral antibiotics (amoxicillin plus clavulanic acid or an equivalent cephalosporin) must be used in acute respiratory exacerbations. -Inhaled antibiotics can be considered for patients with Pseudomonas aeruginosa infection. -All patients with PCD should be recommended annual vaccinations like influenza, pneumococcal, and monoprophylaxis against the respiratory syncytial virus starting in the first year of life.	-In chronic respiratory infections, Inhaled aminoglycoside and beta-lactam antibiotics are recommended. -Dornase-alfa and inhaled bronchodilators both can be used but it is not recommended due to its ambiguous effect. -Long-term patients must visit a pulmonologist and spirometry two to 4 times every year. -Surveillance cultures should be processed in the same manner as cystic fibrosis cultures. -Culture results guide antibiotic therapy in exacerbations, in case of not responding consider additional fungal cultures. -Inhaled corticosteroids and Intravenous immunoglobulin are not recommended. -Lobectomy is not suggested as therapy.
Primary ciliary dyskinesia (16)	2015	Jason Lobo, Maimoona A. Zariwala, Peadar G. Noone	USA / North América	-Daily airway clearance is important with methods like cardiovascular exercise, percussion vests, physical therapy, and positive pressure expiratory valve. -3–7% hypertonic saline has been shown to improve lung function and increase the use of antibiotics in PCD patients. -Inhaled mannitol showed to be better than placebo and improved exacerbations. -In Long-term patients, it is important to do 2–4 revisions of lung function and culture every year.	-Antibiotics should be based on respiratory cultures. -Lung resection may be considered in areas of localized lung disease. -Lung transplants of double lung surgery have good survival outcomes. -It is not recommended Dornase-α because it has been proved to be associated with pulmonary exacerbations. -Inhaled corticoids have not shown any benefit in patients with PCD.
Primary ciliary dyskinesia (PCD): A genetic disorder of motile cilia (20)	2019	Leigh, Margaret W.a; O’Connor, Michael G., Zariwala, Maimoona A.	USA / North América	-Positive Pressure Expiratory devices, vest therapy, manual chest physiotherapy, postural drainage, exercise, and active cycle breathing can be used for mucus clearance of the airway. -Dornase-α is beneficial but can increase exacerbations and decline of lung function in adults. -Inhaled gentamicin has shown a reduction in pulmonary exacerbations, inflammation, and bacterial load; also, chronic macrolides used an improved anti-inflammatory, and anti-quorum sensing reducing exacerbations and improving lung function. -Lobectomy is contraindicated but can be considered in patients with severe localized bronchiectasis, hemoptysis, and bad adherence to treatment.	-Inhaled corticosteroids are not recommended routinely. -Seven percent hypertonic saline improved lung function and a better quality of life. -Systemic antibiotics are effective in the treatment of PCD and should be guided by microbial sputum culture. -Lifestyle change should be promoted by avoiding smoke, immunizations, and infection control practices.
Primary ciliary dyskinesia (15)	2019	Thomas Ferkol; Margaret W. Leigh	USA / North América	-It is fundamental to counsel the family and the patient about this disease. -Enhanced mucus clearance can be facilitated by chest percussion and postural drainage. -It is important to avoid cough suppressants. -Bronchodilators (albuterol) may aid mucus clearance in patients, also it is beneficial the use of anti-inflammatory agents, Dornase-α, and hypertonic saline for better airway clearance. -Lifestyle change is a cornerstone in therapy with avoidance of tobacco smoke, pollution, respiratory irritants, and respiratory pathogens.	-Sputum Gram stain and culture results determine antimicrobial therapy. -Lobectomy is considered in severe localized bronchiectasis or atelectasis, but it has long-term adverse consequences. -Lung transplantation is useful in end-stage lung disease. -Long-term patients should receive routine immunizations against Influenza, S. pneumoniae, and pertussis.
Cuidado respiratorio domiciliario en discinesia ciliar primaria (17)	2021	Ávila, I. J., Cruz Mosquera, F. E., Arango, A. C. y Jiménez Durán, D. P.	Colombia/South América	-All the interventions have the goal of minimizing complications in the patient. -The most important management is respiratory physiotherapy to reduce mucus in the airway. -7% hypertonic saline with beta-2 adrenergic is recommended to reduce bronchial hyperreactivity.	-The use of hypertonic saline at 6% is recommended before physiotherapy increases the sputum expectoration. -A device that increases positive expiratory pressure is used in vias to improve bronchial cleanliness.
Kinesiología respiratoria en niños con disquinesia ciliar primaria (18)	2019	Rodrigo Torres-Castro 1, Klgo. Jordi Vilaró 2, Klgo Homero Puppo	Chile / South América	-Daily airway physical therapy along with antibiotic treatment of infectious exacerbations is2 the cornerstone of treatment for children with PCD.	-Trained professionals must be available who have a thorough knowledge of the disease and have sufficient expertise in the application of the various respiratory physiotherapy manual and instrumental techniques.

The findings from Scopus, Google Scholar, and PubMed indicate that four papers from North America were prominent in the search results. The first article, published in 2016, presents a consensus and recommendations based on American guidelines. It emphasizes the significance of multidisciplinary care for primary ciliary dyskinesia (PCD) and raises questions regarding its implementation. This article is a compilation of research conducted in Canada and other parts of North America.

The second paper, published in 2015, focuses on the use of physical therapy for airway clearance in PCD. It presents the authors' opinions and is specific to North America. This study is notable as it discusses lobectomy as a therapeutic option and mentions the potential use of hypertonic saline solutions at concentrations ranging from 3% to 7%. It also approves the use of mannitol.

The third article, published in 2019, discusses the use of gentamicin to reduce clinical exacerbations in PCD and emphasizes the importance of lifestyle modifications. It also highlights the advantages of using Dornase alfa as a therapy compared to previous treatments.

The fourth article, also published in 2019, focuses on the value of family therapy and discourages the use of cough suppressants in PCD management. It underscores the significance of maintaining a positive outlook on life. This publication is unique to North America and advocates for the use of Dornase alfa and lobectomy in severe cases.

In South America, only one case report and one guide were found during the search. The case report, carried out in Chile in 2019, emphasizes the importance of therapy provided by qualified professionals and subject matter experts (17).

The Colombian guide, published in 2021, is a review with additional comments from the authors. One notable element in this guide is the recommendation to use a 6% hypertonic saline solution before physiotherapy to improve expectoration in patients (18).

No data was available for Central America and the Caribbean. The management information in Latin America mainly relies on case studies, research from North America, and treatment guidelines for other diseases like cystic fibrosis. The lack of innovative PCD research is evident in the Caribbean, Central American, and South American nations.

A comparison between the North American and European guidelines revealed similar information. The most recent therapeutic and diagnostic recommendations come from the American Thoracic Society for Pediatric Pulmonary Recommendations. The European guideline mentions the use of genotyping or the PICADAR questionnaire. The American guideline suggests screening patients with PCD symptoms and their families, while the European guideline recommends evaluating babies with unexplained respiratory distress, males with immotile sperm, and women with recurrent ectopic pregnancies. The American recommendations include nasal nitric oxide as a diagnostic tool for patients aged 5 and above, whereas the European standard proposes its use for those aged 6 and above. As displayed in Supplementary Table S1.

Both guidelines recommend antibiotic treatment, saline solution for airway clearance, and lavage methods for patient care. The American guideline emphasizes physiotherapy and expectoration exercises, while the European guideline highlights lifestyle changes, vaccination, and the avoidance of risky behaviors such as smoking.

## Discussion

This review represents the first comprehensive examination of studies and recommendations specific to Latin America in the context of PCD. The limited research in this region has prompted a need for a study focusing on the diagnosis and treatment of this condition.

PCD significantly increases both mortality and morbidity among young individuals (2). Even those who surpass the age of 18 face poor survival rates and endure persistent respiratory illnesses, leading to a diminished quality of life. Diagnostic techniques commonly employed in North America and Europe are not accessible in underdeveloped countries, particularly those with limited financial resources. Consequently, further research is imperative in underdeveloped countries where there is currently an absence of information regarding the diagnosis or treatment of PCD.

We conducted a summative comparison of the two prominent perspectives on primary ciliary dyskinesia (PCD): the European Respiratory Society and the Thoracic Society of Pediatric Pulmonology (North American). Given the limited availability of information in Latin America papers a direct comparison between the two articles found in our systematic search is not feasible, given their disparate natures. Specifically, one of the Latin American articles assumes the form of a Case Report, inherently limited in its scope to offer extensive insights into patient management. Conversely, the other article represents a guideline grounded in the authoritative expertise of the Thoracic Society of Pediatric Pulmonology, thereby highlighting the paucity of available information in the region.

Nasal nitric oxide measurement is recommended as one of the screening tools by both the American Thoracic Society and the European Respiratory Society. However, in less developed nations of Central and South America, the use of chemiluminescence for this test is unavailable and cost prohibitive. In such settings, alternative options include the use of less expensive portable analyzers that employ electrochemical detection. In developed nations, the PICADAR score emerges as a valuable screening tool. It comprehensively considers diverse factors such prematurity and chest symptoms during neonatal period, admission to neonatal unit, presence of wet cough since early childhood, situs abnormalities, congenital heart defects, persistent rhinitis, chronic ear infections, or hearing problems (10). This comprehensive assessment empowers healthcare providers to gain a deeper understanding of the patient's condition and facilitates the customization of targeted and effective management strategies (10). The absence of these diagnostic techniques poses a challenge when the disease is not considered as a potential condition in young individuals, as there is no clear differentiation in their clinical presentation from other differential diagnoses. Due to the similarity of symptoms with conditions like cystic fibrosis and asthma, the absence of proper diagnostic resources is associated with an increased mortality rate among patients, emphasizing the crucial need for accurate diagnosis.

## Current research gaps

Most of the research conducted in Latin American nations did not meet the inclusion criteria for this review, as it primarily consisted of undergraduate research papers, government statements, or outdated publications. While our search encompassed Spanish and English words, it is important to acknowledge that excluding Portuguese may have resulted in the exclusion of additional relevant articles. Moreover, many countries in the region do not consistently publish their research information in accurate and scientifically rigorous formats, as evidenced by publications found outside of peer-reviewed journals and on websites with minimal or no academic impact.

It is crucial to emphasize that the recommendations provided by the North American and European guidelines, while influential, do not offer a definitive framework for determining the necessity of care, nor do they adequately consider regional variations in the disease and its treatment. This discrepancy becomes particularly evident in other regions, such as Latin America, where the accessibility of appropriate diagnostic tools and treatment resources is limited. The lack of suitable technology and resources further compounds the challenges in achieving a timely and accurate diagnosis, as well as implementing appropriate treatment strategies in these regions.

## Conclusion

In conclusion, the management of primary ciliary dyskinesia (PCD) in Latin America is currently challenging due to the limited research and diagnostic resources available in the region. The lack of established guidelines and diagnostic techniques specific to Latin America necessitates the reliance on information extrapolated from Europe and North America. However, this approach may not be optimal given the regional variations and limitations in accessibility to diagnostic tools.

It is crucial to emphasize the need for international studies that are specifically designed and focused on the Latin American population affected by PCD. These studies should aim to address the unique challenges and considerations of managing PCD in the region. By conducting well-planned research targeted towards Latin America, we can generate valuable insights and recommendations to improve the diagnosis and management of PCD in this population.

Efforts should also be made to enhance collaboration between researchers and healthcare professionals in Latin America and those from more resource-rich regions. This collaboration can facilitate the transfer of knowledge, expertise, and resources, ultimately contributing to improved care and outcomes for PCD patients in Latin America.

Overall, there is a pressing need for further research, increased awareness, and tailored management strategies for PCD in Latin America. By addressing these gaps, we can advance the understanding and treatment of this complex condition, ultimately improving the lives of individuals affected by PCD in the region.
