# Supplementary material for: Primary ciliary dyskinesia diagnosis and management and its implications in America: a mini review
Source: Front Pediatr. 2023 Sep 8;11:1091173. doi: 10.3389/fped.2023.1091173 (PMC10514901; doi:10.3389/fped.2023.1091173)
Supplement: Supplementary file 1 [file Table1.pdf]

Supplementary data

Supplementary table.1 PICADAR Score

PICADAR SCORE SYSTEM		
Does the patient have a daily wet cough that started in early childhood?	Yes-Continue<br>No-STOP	
• Was the patient born pre-term or full term?	Term	2
• Did the patient experience chest symptoms such as tachypnoea, cough, or pneumonia, in the neonatal period?	Yes	2
• Was the patient admitted to a neonatal unit?	Yes	2
• Does the patient have a situs abnormality? (Situs inversus or heterotaxy)	Yes	4
• Does the patient have a congenital heart defect?	Yes	2
• Does the patient have persistent perennial rhinitis?	Yes	1
• Does the patient experience chronic ear or hearing symptoms? (e.g., glue ear, serous otitis media, hearing loss, ear perforation)	Yes	1

Supplementary table 2 Summary of main PCD guidelines

	Diagnosis	Which patients should be tested?	Number of controls per year	Management	General Recommendations
European Respiratory Society	• Nasal nitric oxide in children > 6 years old and suspected adults<br>• High speed videomicroscopy analysis (Ciliary beat frequency and pattern)<br>• Respiratory cells culture improves accuracy<br>• Ciliary ultrastructure analysis by transmission electron microscopy as a part of the diagnostic workup (High specificity)<br>• Immunofluorescence of ciliary proteins is useful in limited resourced settings<br>• Genotyping<br>• PICADAR questionnaire<br>• Radio aerosol mucociliary	• Patients with persistent wet cough, situs anomalies, persistent rhinitis, chronic middle ear disease with or without hearing loss, history in term infants of neonatal upper and lower respiratory symptoms<br>• Patients with normal situs presenting with	• Annual spirometry<br>• Annual High Resolution Computed Tomography (HRCT)<br>• Annual lung clearance index<br>• Cough swab or sputum culture every 3 months	• Prophylactic antibiotics if there is history of repeated courses of antibiotics to treat airways infections<br>• High-dose oral antibiotics are recommended at the first sign of worsening respiratory symptoms or if there is deterioration in lung function<br>• Bronchoalveolar lavage can be considered (left in consideration to the physician)<br>• In unstable patients, consider 3 months of IV antibiotics<br>• Nebulized rhDNase in selected patients	• Regular exercise<br>• Avoidance of cough suppressants<br>• Annual vaccination for pneumococcus and influenza<br>• Avoidance of active and passive smoking

	clearance tests	other symptoms suggesting PCD<br>• Siblings of patients with PCD, particularly if they have symptoms<br>• Babies with unexplained respiratory distress<br>• Males with immotile sperm<br>• Females with recurrent ectopic pregnancy and if there are other features of PCD		• Patients with otitis media and effusion should be treated in a conservative way (regular audiologic evaluation, hearing aids, and audition therapy)<br>• Lobectomy is reserved for advanced bronchiectasis<br>• Airway clearance with saline solution douches.	

American guidelines guideguideThoracic Society – Pediatric Pulmonology	- Evaluation of clinical features<br>- Ciliary biopsy for electron microscopy in children < 5 years old<br>- Nasal nitric oxide in children > 5 years and adults, the test is diagnostic with values < 77 nl/min<br>- High speed videomicroscopy analysis (functional ciliary beat/waveform analysis)<br>- Immunofluorescence testing<br>- Diagnostic criteria vary per age<br>- Radio aerosol mucociliary clearance testing in children > 7 years old	- Patients with clinical features suggestive of PCD.<br>- Siblings of patients with clinical features of PCD.	- Outpatient visits with a pulmonologist experienced in chronic suppurative disease 2-4 times a year.<br>- Surveillance cultures of expectorated sputum or oropharyngeal cough swabs 2-4 times per year<br>- Otolaryngology 1-2 times a year in children. Adults as needed.<br>- CXR performed at diagnosis and during respiratory exacerbations as indicated. In stable	- When patients do not respond to cultured direct antibiotics, consider additional NTM+ fungal cultures, ABPA testing, and bronchoscopy with bronchoalveolar lavage fluid cultures to guide antimicrobial therapy.<br>- Pressure equalization tubes in children with PCD with hearing deficits or speech delay and middle ear effusions.<br>- Daily saline irrigation in patients with chronic rhinosinusitis.<br>- Airway clearance through daily chest physiotherapy.<br>- 2-3 weeks of antibiotics in	- All patients with a diagnosis of PCD should visit at least once a PCD foundation Clinical Center to officially confirm the diagnosis<br>- Long term follow up should be made in a PCD Foundation Clinical Center<br>- Patients undergoing PET should be counselled on the likelihood of multiple insertions, postoperative otorrhea, and possibility of permanent tympanic membrane perforation. These patients should visit the otolaryngologist every 3-6 months

			patient 2-4 times per year<br>- Chest CT recommended at least once after the diagnosis. Some centers perform it every 5 years.<br>- Audiology assessment suggested at diagnosis and subsequent evaluations coordinated with otolaryngology.	case of acute exacerbations (amoxicillin + clavulanic acid/cephalosporin)<br>- Severe exacerbations may require parental antibiotics<br>- In chronic respiratory infections: chronic suppressive inhaled antibiotics (aminoglycosides + beta lactam antibiotics)<br>- Inhaled hyperosmolar agents may be used<br>- DNase (Pulmozyme) is recommended<br>- Inhaled bronchodilators<br>- Lobectomy is not routinely suggested.	while the tube is in place.<br>- Daily cardiovascular exercise<br>- Patients should receive recommended vaccinations per local schedules.<br>- In the first year of life, monthly immuneprophylaxis against RSV in patients who require prolonged oxygen supplementation.
